# On the Anti-Corrosion Property of Dry-Gel-Conversion-Grown MFI Zeolite Coating on Aluminum Alloy

**DOI:** 10.3390/ma13204595

**Published:** 2020-10-15

**Authors:** Shang-Tien Tsai, Wen-Chyuan ChangJean, Lin-Yi Huang, Tseng-Chang Tsai

**Affiliations:** 1 Hawing GemS Technology Corporation, Taichung 402, Taiwan; cuccid@gmail.com (S.-T.T.); wenchuanuni@yahoo.com.tw (W.-C.C.); 2 Department of Applied Chemistry, National University of Kaohsiung, Kaohsiung 811, Taiwan; x313520@yahoo.com.tw

**Keywords:** MFI zeolite film, dry gel conversion, reactor humidity control, zeolite hydrophobicity, anti-corrosion resistance

## Abstract

MFI zeolite film coated on AA6061 alloy was prepared from fumed silica modified with/without n-octyldecyltrimethoxysilane (ODS) by means of dry gel conversion (DGC) method. The DGC-grown MFI zeolite film could form a strong barrier to protect AA6061 surface against the corrosion from NaCl solution. By using fumed silica as a starting material, the hydrophilicity and anti-corrosion capability of the MFI zeolite film declined with increasing humidity in the DGC synthesis. By silanization with ODS, the surface hydrophobicity of the MFI zeolite film increased, leading to substantial enhancement in anti-corrosion capability. On the other hand, MFI film grown from ODS-modified fumed silica exhibited low hydrophilicity and a much improved anti-corrosion protection property by four orders of magnitude, even stronger than the ODS post-treated MFI film. The strong anti-corrosion capability is attributed to the “thick layer” surface hydrophobicity of zeolite crystal.

## 1. Introduction

Hexavalent chromium Cr(VI) has been proven as robust and endurable protection coating of metal to prevent from the attacks by destructively aggressive environmental components such as acid, base and chloride. Cr(VI) has been widely used in the pre-treatment and primer layers of metal. Due to carcinogenic concern, RoHS (Restriction of Hazardous Substances) has regulated the application of Cr(VI) coating. Currently trivalent chromium pre-treatment is used as the main substitution of Cr(VI) coating. Although non-chromate coating is the most environmentally-friendly and desirable alternative, only very few Cr-free coatings have been proven as a practical solution [1]. Strong metal protection could be provided by corrosion inhibitors, physical barriers, and galvanized substances. Recently some multifunctional composite coatings encompassing low porosity [2], zigzagging diffusion path [3,4,5], self-healing capability [6], and hydrophobicity [7] have been proposed as the potential substitution of the traditional Cr(VI) coating.

Some of the alternatives of Cr(VI) coating include sol-gel, low temperature cationic plasma deposition, ceramer coatings, various inorganic and organic inhibitors and conducting polymers. Among which the sol-gel chemistry is useful for the fabrication of organic, inorganic and organic-inorganic hybrid coatings with strong protection of metal [8,9]. By sol gel chemistry, many organic-inorganic network of Si, Ti, Zr, Al, Fe, B, etc. could be generated from monomeric metal or metalloid alkoxide precursors M(OR)n where M represents a network-forming element, and R is typically an alkyl group. The sol gel route has the advantages in manipulating the microscopic properties, such as molecular structure and weight, and also macroscopic properties such as viscosity, strength, and toughness. Therefore, satisfactory properties of the anti-corrosion film could be designed rigorously by sol gel chemistry. Nevertheless, sol-gel process usually entails high curing temperature that is not compatible with many metal alloys.

Zeolite film has been proven as an effective anti-corrosion coating for the protection of various metals from corrosion, particularly useful in severe corrosive environments in strong acid and base environments (pH 3–12) and in high temperature [10,11,12]. Cai and Yan summarized the exploratory works of corrosion-resistant zeolite coatings [13]. Most recently, Calabrese et al. published another comprehensive review [14]. Zeolite film are used for anticorrosion applications by either passive or active mechanism. The approach by passive mechanism is to keep the structure-directing agent (SDA) intake inside zeolitic micropore. By doing so, zeolite film could be defect-free and gas-tight to function as an efficient corrosion-resistant barrier to protect metal surface from the attack of aggressive species [13,15]. On the other hand, the approach by active mechanism is to release a corrosion inhibitor from zeolite micropores. Dias et al. [16] used zeolite microparticle acting as the reservoir for Ce(III) in silica–zirconia sol–gel films. The local pH change in solution during corrosion process would activate the release mechanism of Ce(III) from zeolite microparticle. Then Ce(III) would precipitate in the cathodic areas to stop corrosion.

Zeolite could be deposited on metal surface by direct crystal growth method with in-situ crystallization [17,18], dry-gel conversion (DGC) [10,11], secondary growth [19], zeolite composite coating [16,20], or self-assembly of zeolite crystal [21]. By direct crystal growth method, the strong anti-corrosion property of a zeolite coating changes with the preparation procedure in a complicated and divergent way [22,23,24]. Yan’s group has demonstrated the applicability of high-silica-zeolite MFI (International Zeolite Association code) coating for providing corrosion resistance of various metal surfaces, particularly for aluminum alloy such as AA- 2024, 5052, 6061, and 7075 [13,17,18]. Liu et al. developed a self-assembly method to prepare b-oriented MFI monolayers from sec-butanol-modified zeolite microcrystal—sec-butanol mixture through a thin layer of water as a temporary soft substrate [21]. The self-assembly method could be applied to various substrates, such as glass, stainless steel and aluminum, in regardless of surface roughness.

The hydrophobicity of zeolite film is one of the key properties in multi-functional smart coating for many applications, such as corrosion resistant coating [14]. Hydrophobic zeolite could be prepared through co-condensation of organo-functional silane with inorganic silica precursors [25], from the starting material of low aluminum content [26,27,28], hydrothermal synthesis in F^-^ medium [29]. On the other hand, the surface property of zeolite film could be modified by several post synthesis methods, such as grafting of organo-functional silane onto the pore walls with versatile silanes bearing wide ranges of organic groups such as amine, phenyl, thiol, isocyanate, and urea. Kosinov et al. reported that the MFI membrane by surface modification with triethoxyfluorosilane showed significant improvement in hydrophobicity and recovery of ethanol from ethanol/water mixtures by pervaporation [30]. Yan et al. reported that the hydroxyl groups of MFI film vanish after vapor phase silylation with chlorotrimethylsilane, leading to improved hydrophobicity [31].

While the corrosion resistance of MFI zeolite film could be improved by increasing hydrophobicity, the difference in the effect of hydrophobicity from different origins on the corrosion resistance has been rarely studied. The present paper studies the effect of silica precursor in DGC method on the surface property and corrosion protection property of MFI zeolite film. The anti-corrosion properties of DGC-grown MFI zeolite film and post modified hydrophobic MFI zeolite film are compared.

## 2. Experimental

### 2.1. Substrate Pre-Treatment Procedure

AA6061-T6 aluminum alloy (Aluminium Corporation, Taiwan) was cut into 2 × 2 × 0.1 cm pieces and used as the substrate. The polished metal piece was further immersed in 30% H_2_O_2_ (Wako, Japan) for 45 min for oxidation of surface aluminum; or in 1 N NaOH (97%, Wako, Japan) solution for 30 min for cleaning the surface, then rinsed with distilled water.

### 2.2. Dry-Gel-Conversion (DGC or Steam Assisted Crystallization of Dry Precursor Film)

Dry gel conversion (DGC) method was used to prepare the MFI zeolite coated AA6061 metal plate. The DGC method is a two-step process. The first step is to coat precursor gel on the surface of 6061 Al alloy substrate following with a drying treatment. The second step by “DGC” process is to seal the zeolite precursor gel coated AA6061 substrate inside a hydrothermal reactor. The whole hydrothermal reactor was kept at a hydrothermal temperature for a certain time period. The detail experimental procedure is described below.

A hydrophobic silica precursor denoted as OSF was prepared by refluxing of dry toluene solution of fumed silica (5 g) and n-octyldecyltrimethoxysilane (0.98 mL, 96%, Sigma-Aldrich, St. Louis, MO, USA) under nitrogen at 80 °C for one day.

The DGC preparation procedure for growing MFI film on AA6061 substrate was reported [10]. Two silica sources denoted as 75F and 75OSF were prepared by mixing 25 mol% tetraethyl orthosilicate (TEOS, 98%, Sigma-Aldrich, St. Louis, MO, USA and 75% fumed silica or OSF, respectively, for the synthesis of MFI zeolite film. For example, a mixture of 1.0 mol silica, 0.25 mol tetrapropyl ammonium hydroxide (40 wt% TPAOH, Tritech catalyst & Intermediate Pvt. Ltd., India) and 20 mol H_2_O was stirred until it becomes a homogenous solution. A small quantity of Tween-20 (polyoxyethylene 20 sorbitan mono stearate, Tween-20/SiO_2_ = 0.01 wt/wt) was added to improve the wetting of the substrate. The final precursor sol remained stable for over five weeks at room temperature.

The above precursor sol was deposited on the substrate by dip coating (5-min immersion and 2.4 cm min^−1^ withdrawing rate). The coated substrate was placed on top of a stainless steel frame in a 125 mL polytetrafluoroethylene (PTFE) cup containing *z* mL of distilled water. The whole setup was enclosed inside an autoclave at 180 °C for 12 h. The film produced was denoted as 75OSF- or 75F- w*z* when fumed silica or ODS-modified silica was used with TEOS mixture.

### 2.3. Characterization of Zeolite Film

X-ray diffraction patterns of the zeolite films were taken with the Rigaku Multiflex model 2 kW X-ray diffractometer using Cu Ka radiation at 40 kV and 30 mA. In reference to one MFI zeolite powder sample, the relative crystallinity was determined semi-qualitatively as the relative intensity of the diffraction peaks.

The zeolite films were examined for morphology with a HITACHI S-3400N scanning electron microscopy (SEM, HITACHI, Japan) at 15 kV operating voltage and 10 mm working distance. All samples were submitted to intense ultrasonic radiation before measurement. The film sample for cross-sectional SEM imaging was etched by dipping the M12 samples in 49 wt % HF for 5 s.

Sessile drop method was applied for the determination of contact angle of MFI zeolite film. General Type Phoenix 300/150 static contact angle analyzer supplied by Surface Electro Opics (SEO, Korea) was used. The contact angle was measured on the distiller water with drop size of 10 μL assuming spherical drop shape.

### 2.4. Electrochemical Property Measurement

The room-temperature corrosion behavior of various AA6061 plates coated with/without MFI zeolite films in 0.5 M (3 wt %) NaCl aqueous solution was investigated with a model CHI-627D electrochemical spectroscope (CH Instrument, TX, USA). A saturated calomel electrode (SCE) was used as the reference electrode, while a platinum wire served as the auxiliary electrode. The working electrode was either a bare substrate or one coated with MFI zeolite film. Both were immersed 0.78 cm^2^ into the solution limiting to the surface of the center of the sample by o-ring seal. The potentiostatic polarization spectrum (Tafel plot) was taken with d.c. polarization scan, beginning at 2 V vs. SCE with a stabilization time of 1 min, and continuously in the anodic direction at 5 mV s^−1^. The corrosion potential (E_corr_) was determined from the cathodic current curve and the anodic current curve of the Tafel plot fitted with the Butler–Volmer equation numerically. The corrosion current (I_corr_) was determined with Tafel extrapolation technique as the intersection of tangent line of anodic or cathodic polarization curve at ±50 mv of E_corr_.

## 3. Results

### 3.1. Effect of Water Usage on the Hydrophilic Film Structure of Zeolite Coated 6061 Al Alloy

Dry gel conversion (DGC) method was used to grow MFI zeolite film on top of metal surface. The “DGC” process is to “in-situ” crystallize the *amorphous* dry precursor into *crystalline* MFI zeolite film in the presence of steam. Notation of 75F-w*z* for a MFI zeolite film was used to represent the hydrothermal synthesis of zeolite film from 75% fumed silica—25% TEOS mixture at 180 °C for 12 h using water reservoir of *z* mL. By such process, the MFI zeolite film thickness could be controlled in terms of precursor layer thickness and DGC process condition.

**Figure 1:**
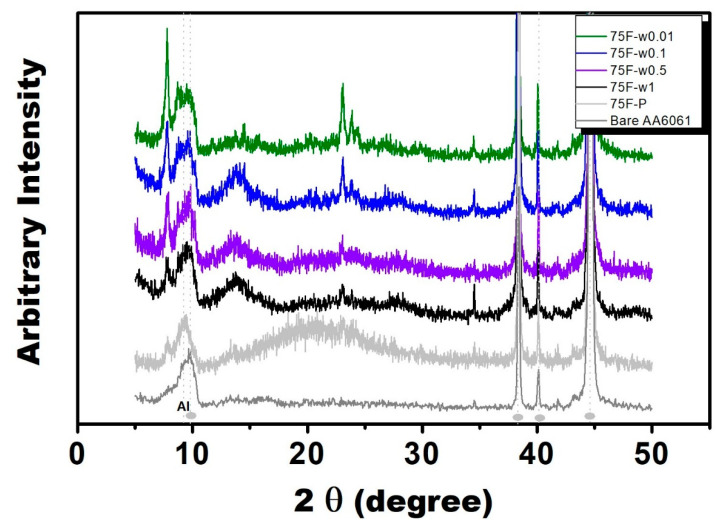
X-ray diffraction patterns of bare and zeolite coated AA6061 aluminum alloy (Synthesis gel: 2.88 SiO_2_ (0.72 TEOS + 2.16 fumed silica): 0.74 TPAOH: 64.4 H_2_O; Dry gel condition: 180 °C, 12 h, water amount in cm^3^ referring to the legend of 75F-w*z*).

The precursor gel 75F-*P* (zeolite precursor gel) revealed weak and broad characteristic x-ray diffraction pattern, which can be attributed to nano crystallite as a result of Scherrer equation. Four DGC-grown films 75F-w*z* were prepared through DGC process using various water amounts (*z* ml). X-ray diffraction spectroscopy was used to examine MFI zeolite film growth. As shown in Figure 1, the DGC-grown films using very low water amount, 75F- w0.01 and -w0.1 both revealed characteristic main diffraction peaks of MFI zeolite at 2θ of 7.7°, 8.5°, 23.0°, 23.9°, 34.4° (in reference to JCPDS-ICDD Card No. 37-0361), indicating crystalline zeolite MFI structure. On the other hand, the MFI zeolite films 75F- w0.5 and -w1.0 DGC-gown in high water amount revealed weak X-ray diffraction intensity as an indication of low crystallinity.

**Figure 2:**
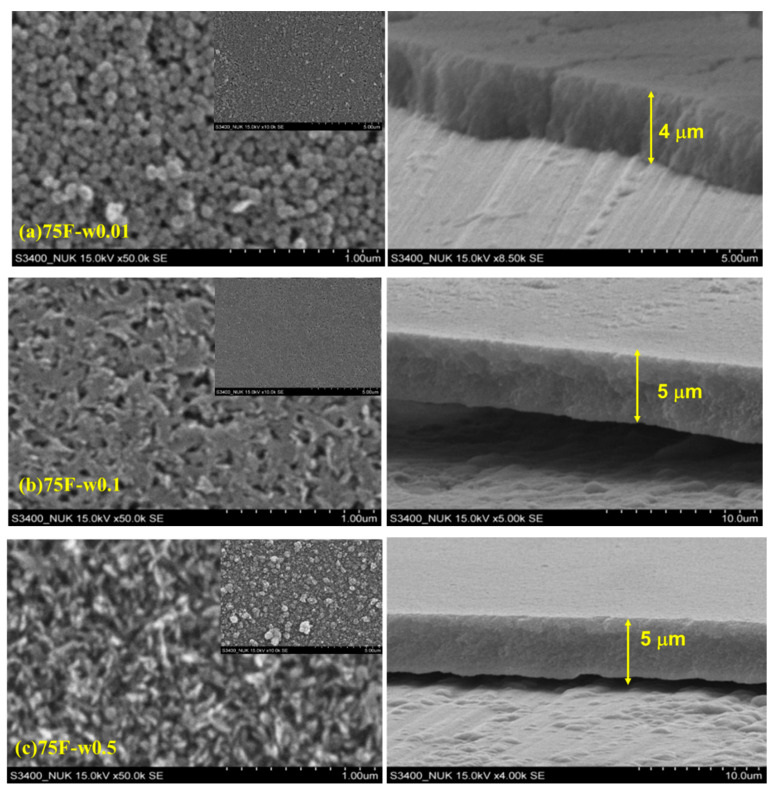
SEM images at different magnification scales of various DGC-grown MFI films; projection: Large magnification ×50,000 and small magnification ×10,000 (inset); and cross-section; (**a**) 75F-w0.01 projection and cross-section (×8500); (**b**) 75F-w0.1 projection and cross-section (×5000); (**c**) 75F-w0.5 projection and cross-section (×4000).

As shown in Figure 2, different DGC-grown MFI zeolite films had different morphology. By increasing water usage during DGC, the morphology of 75F- series changed from nanoparticle of 100 nm (-w0.01 film) to sheet-like (-w0.1) intergrowth and rice shape (-w0.5) with film thickness of 4, 5 and 5 μm, respectively. All the cross section images indicated that the coating films of 75F- series were in close and dense packing. Strong adhesion of MFI zeolite film on AA6061 surface was observed with tape test.

### 3.2. Effect of Water Usage on the Hydrophobic MFI Zeolite Coating

Contact angle of MFI zeolite films was determined to examine its hydrophobicity. As shown in Table 1, while the contact angle of bare AA6061 substrate was 73°, all the DGC-grown 75F-w*z* films coated AA6061 became hydrophilic having contact angle of 27–29° and <9° for 75F-w0.01 and -w0.1; and 75F-w0.5 and -w1.0 films, respectively. The hydrophilic 75F-0.5w film was subjected to silane treatment by which the MFI zeolite film became hydrophobic having contact angle of 141°. As comparing to the as-synthesized 75F-0.5w film having contact angle of <9°, the silane modified 75F-w0.5-ODS turned into hydrophobic nature.

**Figure 3:**
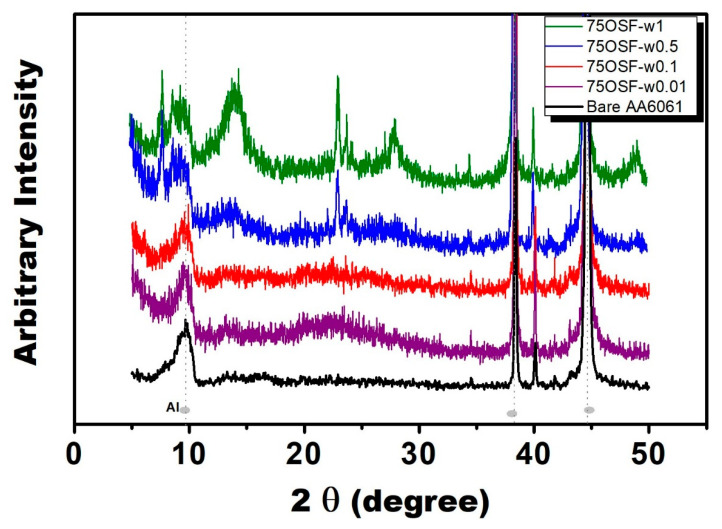
X-ray diffraction patterns of bare and zeolite coated AA6061 aluminum alloy (Synthesis gel: 2.88 SiO_2_ (0.72 TEOS + 2.16 ODS modified fumed silica): 0.74 TPAOH: 64.4 H_2_O; Dry gel condition: 180 °C, 12 h, water amount in cm^3^ referring to the legend of 75OSF-w*z*).

**Figure 4:**
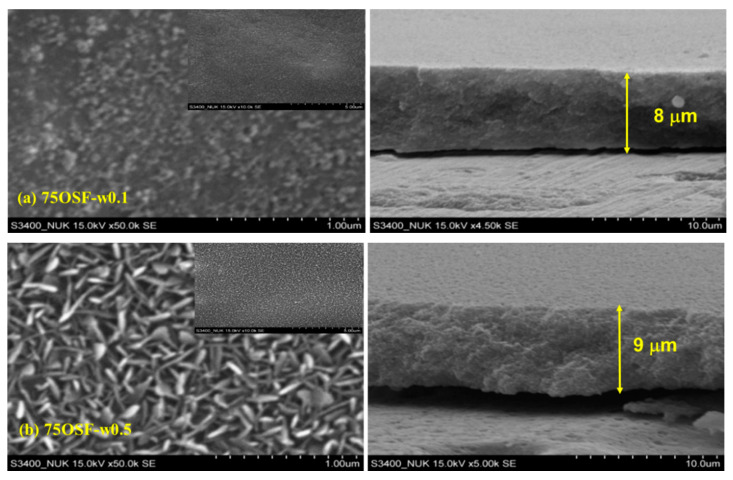
SEM images at different magnification scales of various DGC-grown MFI films; projection: Large magnification ×50,000 and small magnification ×10,000 (inset); and cross-section; (**a**) 75OSF-w0.1 projection and cross-section (×4500); (**b**) 75F-w0.5 projection and cross-section (×5000).

By using water amount of 0.5 mL, DGC-grown 75OSF-w0.5 revealed characteristic diffraction patterns of MFI zeolite (Figure 3). Further increase water usage in the DGC process (75OSF-1w) led to formation of impurity phase. The crystal in the MFI zeolite film was in uniform rod morphology (Figure 4); and highly hydrophilic with contact angle of <9°. The other two samples 75OSF- w0.01 and w0.1 preparation using small water amount of 0.01 and 0.1 mL exhibited slight hydrophobicity (contact angle of 56–68°) but did not appear characteristic MFI zeolite diffraction pattern. The MFI zeolite film was consisted of nano crystal (Figure 4).

### 3.3. Protection of Zeolite Coated 6061 Al Alloy Substrate against Salt Corrosion

**Figure 5:**
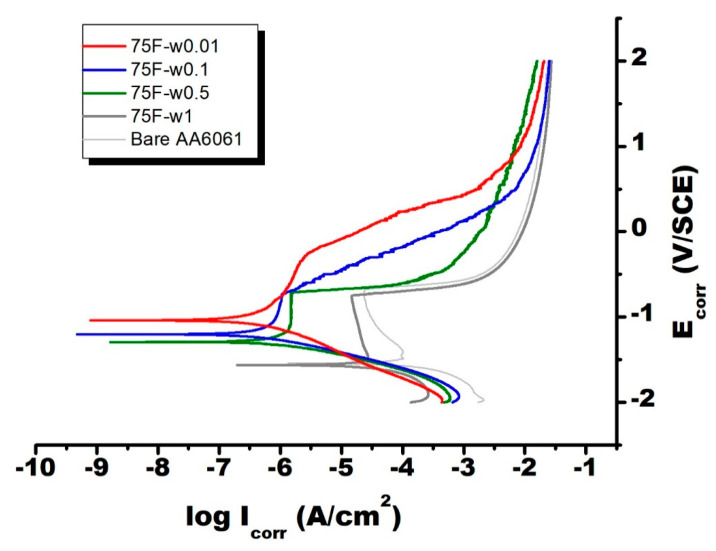
Electrochemical polarization spectroscopy of bare and dry gel conversion (DGC)-grown 75F- coated AA6061. Measurement was done while immersing in 3 wt % NaCl solution.

**Figure 6:**
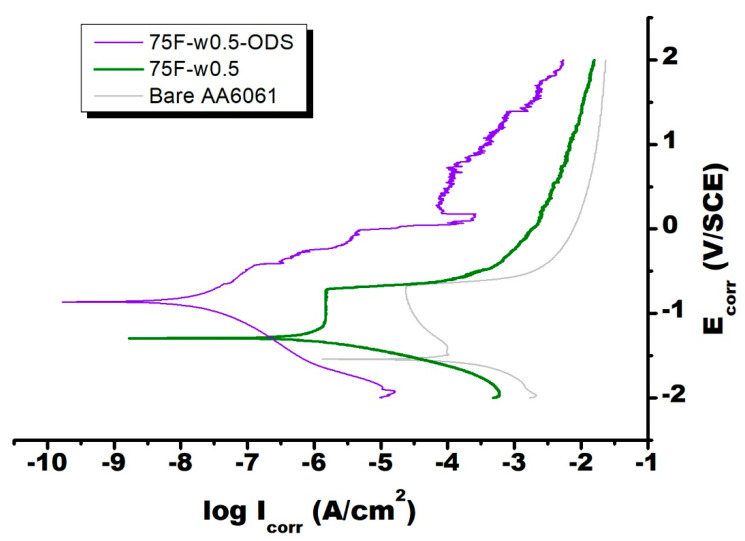
Electrochemical polarization spectroscopy of bare, 75F-w0.5 and ODS modified 75F- w0.5 film coated on AA6061. Measurement was done while immersing in 3 wt % NaCl solution.

**Figure 7:**
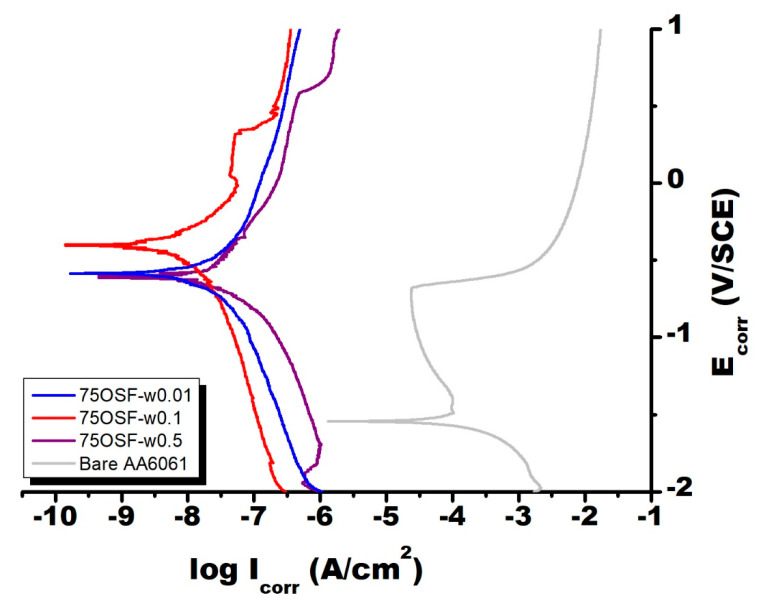
Electrochemical polarization spectroscopy of bare and DGC-grown 75OSF- coated AA6061. Measurement was done while immersing in 3 wt % NaCl solution.

**Figure 8:**
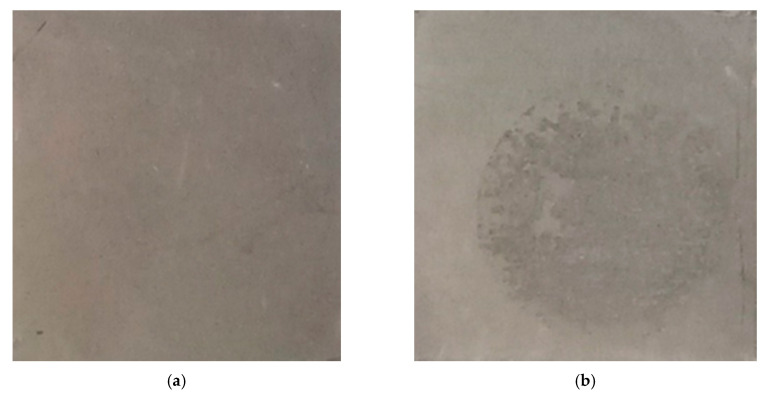
Images of coating before (**a**) and after (**b**) electrochemical polarization test. Measurement was done while immersing in 3 wt % NaCl solution.

As shown in Figure 5, Figure 6 and Figure 7, the electrochemical polarization spectroscopy of different MFI zeolite coated AA6061 alloy plate was measured in 3 wt % NaCl solution. As one example, the surface of the coating 75F-w0.5 partially corroded after the electrochemical polarization test (Figure 8). The visual appearance of the samples along with the polarization test result all indicated the strong corrosion resistance of MFI zeolite film 75F-w0.5. During electrochemical polarization test, the anodic and cathodic reactions are kept in balance in complying with the potential of the metal substrate. In the polarization curve, the total current is the combination of the anodic and cathodic currents. As such, the current from each half reaction changes with the electrochemical potential of the metal. The corrosion potential (E_corr_) and corrosion current (I_corr_) were determined from the cathodic current curve and the anodic current curve of the Tafel plot fitted with the Butler–Volmer equation numerically. The E_corr_ and I_corr_ values both would represent the corrosion resistance of the MFI zeolite coating. Strong anti-corrosion capability of zeolite coating would be indicated by a more positive E_corr_ value and lower I_corr_ value.

Table 1 presents the corresponding parameters of Tafel plot, such as E_corr_, I_corr_, R (polarization resistance), of the zeolite-coated samples grown with different protocols. The I_corr_ of most DGC-grown MFI films were in the range of 10^−5^–10^−9^ A/cm^2^. In reference to the bare alloy plate (I_corr_ = 10^−3.8^ A/cm^2^), the DGC-grown MFI films could reduce the I_corr_ of AA6061 substrate by 1–5 orders of magnitude. Their electrochemical polarization curves had similar shape with the bare AA6061, indicating their anti-corrosion capability mainly came from passivation behavior. Clearly, the ODS modification on MFI film, 75F-w0.5-ODS, made the zeolite film highly hydrophobic with contact angle increased to 141°. Moreover, the ODS modified MFI film, 75F-w0.5-ODS, strengthened significantly the corrosion resistance of the DGC-grown zeolite film, 75F-w0.5-ODS (Figure 6). Therefore, hydrophobicity of MFI zeolite film can improve its corrosion resistance.

As for the 75F- zeolite film series, except 75F-w1.0, the E_corr_ was in the vicinity of 1.2 V/SCE and I_corr_ in the range of 10^−6^–10^−7^ A/cm^2^. Comparing to the E_corr_ of AA6061 at −1.54 V/SCE, the zeolite coated AA6061 had a positive shift of electrical potential of the bare AA6061 by 300–500 mV. The zeolite coated AA6061 showed a reduction of I_corr_ by two–three orders of magnitude. The resulting more noble electrical potential and reduction of corrosion current both indicated clearly the enhancement in corrosion resistance by the zeolite coating. Interestingly, the anti-corrosion capability of MFI film became stronger with increasing crystallinity. Due to low zeolite crystallinity, 75F-w1.0 coating having about the same E_corr_ and marginally reduced I_corr_ with the bare AA6061 substrate fail to provide additional protection capability.

As indicated by the low contact angles smaller than 29°, all the DGC grown 75F- films exhibited hydrophilic nature. It seems that by increasing hydrothermal reactor humidity (by increasing the water usage in the DGC process), MFI film turned into more hydrophilic with negative shift of E_corr_ and increases in I_corr_, as a clear indication of deteriorated corrosion resistance. The 75F-w1.0 film possessed only marginal improvement over the bare AA6061. On the other hand, the silane modified 75F-w0.5-ODS coating exhibited very hydrophobic property with contact angle of 141°, leading to a much more positive E_corr_ by about 700 mV and reduced I_corr_ by four orders of magnitude. Indeed, the anti-corrosion capability of zeolite coating is strongly associated with film hydrophobicity, which could be effectively improved by silane modification.

Alternatively, the 75OSF- zeolite film series were prepared from hydrophobic silica (ODS-modified fumed silica). The 75OSF- serial coatings exhibited higher contact angles and less hydrophilicity than the 75F- serial coatings. Notice that the 75OSF- serial coatings, prepared at different hydrothermal reactor humidity, all exhibited about the same E_corr_ value in between −0.4 and −0.6 V/SCE with an I_corr_ of around 10^−8^ A/cm^2^. Comparing to the E_corr_ of −1.54 V/SCE and I_corr_ of 10^−3.84^ A/cm^2^ of the bare AA6061, the MFI zeolite film grown from ODS-modified silica all exhibited a largely more positive E_corr_ and very low I_corr_ value, as an indication of very strong and effective anti-corrosion coating. The 75OSF-w0.1 exhibited the strongest protection capability with the most positive potential of −0.40 V/SCE and I_corr_ of 10^−8.24^ A/cm^2^.

## 4. Discussion

The corrosion potential and current are usually determined from electrochemical polarization curve (Tafel plot). For the 75F- coating series growing with about a same film thickness of 5 μm, their corrosion resistances were different from each other. As shown in Table 1, the precursor coated AA6061 failed to provide any protection. In comparison, MFI zeolite coating formed a strong protection layer for aluminum alloy AA6061. The strong protection capability of the MFI zeolite film grown from precursor through DGC process could attributed to zeolite MFI structure. In the 75F-wz series, 75F-w0.01 exhibited the maximum corrosion resistance with E_corr_ of −1.04 V/SCE and I_corr_ of 10^−6.73^ A/cm^2^, which could be due to its strongest crystallinity and hydrophobicity. With increasing water usage in the DGC process, the crystallinity of MFI zeolite film weakened with increasing hydrophilicity, and the corrosion resistance deteriorated. The deteriorated protection capability could be due to decreasing zeolite crystallinity and hydrophobicity. As a result, 75F-w1.0 film was very hydrophilic and could provide only marginal protection to AA6061 surface with about the same E_corr_ and a borderline reduced I_corr_ number.

Tafel plot recording potential—current density dependence could provide some valuable insight about the origin of the strong barrier function in MFI zeolite film. The Tafel plot measurement consists of three steps, the Volmer, Heyrovsky, and Tafel step. Among which the former two steps are electron transfer reactions, the latter step does not deal with electron transfer. The Tafel plot could be used to identify the rate determining steps in electrocatalytic reaction. According to Shinagawa et al. [32], if Heyrovsky step is the rate determining step, Tafel slope is dependent on the surface coverage, two Tafel slopes appears at low and high (θ_H_ > 0.6) surface coverage region. If the Tafel step is dominant, the potential—currents dependence is originated from the number of active surface area and coverage, and corrosion potential does not affect the corrosion current. As shown in Figure 5, at low overpotential, the Tafel relationship was followed, showing that both anodic and cathodic reactions are active surface-controlled. At higher overvoltage, a limiting diffusion current appeared on the anodic and cathodic polarization curves as an indication of concentration polarization, in which the transport of chloride ions towards the electrode surface became the rate-determining step.

As shown in Tafel plot, a passive–active transition was observed in bare AA6061 and 75F-w0.5 and -w1.0 films, as an indication of pitting corrosion [33]. The bare AA6061 showed a typical passive behavior with passivation region. The I_corr_ kept stable at about 10^−4.4^ A/cm^2^ in the E_corr_ range from −1.5 to −0.7 V/SCE, indicating that Tafel step is dominant. A characteristic pitting potential (E_p_) of 0.692 V with E_corr_ of −1.54 V/SCE were derived from Tafel plot. The 75F-w0.5 and w1 film exhibited similar passivation region as the bare AA6061, but positive shift of E_p_ and E_corr_ were observed at −0.753 V and −1.56 V/SCE; −0.719 V and −1.29 V/SCE, respectively_._ The Tafel curve of 75F-w0.1 film was different, and its E_p_ could be assigned around −0.728 V with E_corr_ of −1.20 V/SCE. As for 75F-w0.01 film, E_corr_ shifted toward more noble direction to −1.04 V/SCE without any observable pitting potential.

The pitting corrosion of aluminum involves the adsorption and absorption of chloride ions on the oxide film surface of AA6061 and also, the chemical reaction of the oxide layer with chloride ions. The migration of chloride ions from the electrolyte solution through the oxide film or chemisorption of chloride ions onto the oxide surface results in the dissolution of alumina oxide film via the formation of oxide–chloride complexes [34]. As Tafel plot suggested that the improved anti-corrosion property of MFI zeolite film is arisen primarily from barrier mechanism to protect the AA6061 from the aggressive species such as Cl^-^ ion. In practice, aluminum metal forms an oxide layer on its surface. If local area of the passive film break down, leading to significant metal corrosion to occur in a small area to cause pitting corrosion.

The ODS modified 75F-w0.5-ODS with a contact angle of 141° showed a significant shift of E_corr_ toward more positive direction, and a reduced I_corr_ by 3 orders of magnitude. The polarization curve shows that at higher overvoltage, the anodic curve was equally polarized with cathodic curve. Comparing to the polarization curve of hydrophilic film 75F-w0.5, the anodic curve exhibited a more improved anti-corrosion property than the cathodic curve. Therefore, the improved corrosion resistance with improved breakdown potential in the ODS modified 75F-w0.5-ODS could be attributed mainly to the strengthening protection in cathode associated with improved hydrophobicity.

Surprisingly, 75OSF- series coatings all exhibited very strong anti-corrosion properties with very positive E_corr_ and low I_corr_ (Table 1). The 75OSF- films were prepared with DGC process using hydrophobic silica OSF as the silica source. With increasing water usage in the DGC preparation, the crystallinity increased and hydrophobicity decreased. The 75OSF-w0.1 film exhibited the strongest anti-corrosion property with the most positive E_corr_ of −0.40 V/SCE and the lowest I_corr_ of 10^−8.24^ A/cm^2^. While 75OSF-w0.01 film exhibited less protection due to its lower crystallinity, 75OSF-w0.5 film was weaker because of increasing hydrophilicity. Therefore, hydrophobicity and crystallinity of MFI zeolite film are beneficial to corrosion resistance.

Interestingly, the direct-synthesized hydrophobic MFI zeolite film prepared from ODS-modified silica (75OSF- series) has superior corrosion resistance than the hydrophobic ODS-modified MFI film (75F-w0.5-ODS). Notice that all the 75OSF-MFI zeolite film had lower contact angle than the ODS-modified MFI zeolite film. It is known that contact angle could be affected by physical properties such as surface roughness, physical shape of powders, porosity [35,36,37]. Here we applied the prevalent sessile drop method in the determination of contact angles of MFI zeolite films. The DGC transformation method in using different silica raw material might result in varying physical parameters such as porosity. Accordingly, contact angle could not be able to reflect the “intrinsic” hydrophobicity of zeolite film. Some more in-depth measurement methods could be helpful. Presumably, by using the ODS-modified silica, the 75OSF- series zeolite film would be intrinsic hydrophobic as a whole. It is proposed that the 75OSF- series was consisted of hydrophobic zeolite crystal. Accordingly, the zeolite crystal formed strong adhesion interface with the AA6061 surface to form a hydrophobic barrier. Calabrese et al. found that a hydrophobic interface can reduce the interaction between water and metal surface and depress the corrosion processes [38]. In comparison, the ODS-modified zeolite film, 75F-w0.5-ODS, possessed hydrophobic surface. The barrier in 75OSF- series was stronger than that on the surface of 75F-w0.5-ODS. Thus, 75OSF- series had stronger corrosion resistance.

In this context, we have demonstrated that DGC process is a versatile and effective process for preparing zeolite coating with high surface coverage on metal surface for anti-corrosion protection. Matsukata et al. reported that steam is indispensable during the DGC process [39]. Presumably, steam must participate the DGC process for the solid state crystallization of zeolite precursor. They remarked that the effect of steam on the DGC crystallization is rather complicated. Indeed, intriguing effect of steam partial pressure on the DGC crystallization was observed in the present study. By increasing water usage during DGC process, the crystallinity of 75F- coating series decreased (Figure 1) but the crystallinity of 75OSF- increased (Figure 3). Moreover, by increasing water usage, the contact angle of the DGC grown MFI zeolite film all decreased. On the other hand, in terms of anti-corrosion property, the optimum water usage during DGC transformation in using starting material by choosing hydrophilic fumed silica (75F- series) or hydrophobic ODS modified silica (75OSF- series) was 0.01 mL (low usage) and 0.5 mL (high water usage), respectively.

DGC process is a solid state crystallization process in the presence of steam vapor. During the DGC process, the amorphous dry solid gel crystallizes directly into zeolite structure in solid state phase. In principle, during the solid state transformation, the amorphous precursor would go through a series of re-structuring reactions including depolymerization, condensation, oligomerization, polymerization, and crosslinking [40,41]. Along with the precursor transformation, zeolite crystallization proceeds by way of nucleation for nuclei formation and then crystal growth. Among the various reactions, whereas depolymerization generate water, condensation and cross-linking reactions consume water. Presumably depolymerization and nucleation rates would be accelerated by steam. On the other hand, during crystal growth, the extent of silica polymerization and crosslink increases in accompany with increasing water formation. In light of chemical equilibrium, crystal growth could be inhibited by excess water. As a result, steam partial pressure would have different effects on nucleation and crystal growth rate. Therefore, DGC transformation is sensitive to precursor micro-structure. The difference in the optimum DGC water usage between the hydrophilic 75F- series and hydrophobic 75OSF- series could be due to the difference in pore volume, water/steam adsorption capacity of different silica types.

## 5. Conclusions

MFI zeolite film coated AA6061 surface was prepared by dry gel conversion (DGC) method. The DGC-grown MFI zeolite film coated on AA6061 exhibited strong corrosion resistance against NaCl corrosion by means of barrier anti-corrosion mechanism. It was found that hydrophobicity of silica precursor and water amount used in DGC affect significantly the corrosion resistance of the MFI zeolite film. In terms of maximum corrosion resistance, the optimum DGC water usage in the crystallization of hydrophobic silica precursor was found to be higher than that used for hydrophilic silica precursor. The difference in the optimum DGC water usage could be due to the difference in pore volume, water/steam adsorption capacity of different silica types. Comparing to the E_corr_ of −1.54 V/SCE and I_corr_ of 10^−3.84^ A/cm^2^ of the bare AA6061, the MFI zeolite film grown from ODS-modified silica exhibited a largely more positive E_corr_ up to −0.59 V/SCE and very low I_corr_ value down to 10^−8.24^ A/cm^2^, indicating a significant enhancement in corrosion resistance by four orders of magnitude.

Tafel plot recording potential—current density dependence was used to elucidate the origin of the strong barrier function in MFI zeolite film. At low over-potential, the Tafel relationship was followed, showing that both anodic and cathodic reactions are active surface area-controlled. At higher overvoltage, a limiting diffusion current appeared on the anodic and cathodic polarization curves as an indication of concentration polarization, in which the transport of chloride ions towards the electrode surface became the rate-determining step.

## Figures and Tables

**Table 1:** Corrosion parameters derived from the polarization curves of different coated method in 3 wt % NaCl solution.

Sample ID	Contact Angle (°)	Thickness (μm)	E_corr_ (V/SCE)	Log I_corr_ (A/cm^2^)	Rp (Ω cm^2^)	Corrosion Rate (mpy)
Bare AA6061	73 ± 1	0	−1.54	−3.84	4 × 10^2^	76.5
75F-w0.01	29 ± 1	4	−1.04	−6.73	2 × 10^5^	9.9 × 10^−2^
75F-w0.1	27 ± 1	5	−1.20	−6.49	1 × 10^5^	1.7 × 10^−1^
75F-w0.5	<9	5	−1.29	−6.10	4 × 10^4^	4.2 × 10^−1^
75F-w1.0	<9	5	−1.56	−4.48	1 × 10^3^	17.7
75F-w0.5-ODS	141 ± 1	5	−0.81	−8.28	5 × 10^6^	2.8 × 10^−3^
75OSF-w0.01	68 ± 1	8	−0.59	−7.99	4 × 10^6^	5.4 × 10^−3^
75OSF-w0.1	56 ± 1	8	−0.40	−8.24	8 × 10^6^	3.1 × 10^−3^
75OSF-w0.5	<9	9	−0.62	−7.54	2 × 10^6^	1.6 × 10^−2^
